# Role of sarcoplasmic reticulum junctional proteins in skeletal muscle strength

**DOI:** 10.1186/1471-2253-14-S1-A19

**Published:** 2014-08-18

**Authors:** Barbara Mosca, Osvaldo Delbono, Maria Laura Messi, Leda Bergamelli, Mirko Vukcevic, Ruben Lopez, Susan Treves, Miyuki Nishi, Hiroshi Takeshima, Francesco Zorzato

**Affiliations:** 1 Department of Life Sciences and Biotechnology, General Pathology section, University of Ferrara, Ferrara, 44100, Italy; 2 Department of Physiology and Pharmacology, Wake Forest University School of Medicine, Winston-Salem, N.C. 27157, USA; 3 Departments of Biomedicine and Anesthesiology, Basel University Hospital, Basel, 4031, Switzerland; 4 Department of Biological Chemistry, Graduate School of Pharmacological Sciences, Kyoto University, Tokyo, 113-0033, Japan

## Background

Skeletal muscle constitutes approximately 40% of body mass, and age-induced decrease of muscle strength impinge on daily activities and on normal social life in the elderly. Loss of muscle strength has been recognised as a debilitating and life threatening condition also in cachexia in cancer patients and in clinical conditions associated with prolonged bed rest. Skeletal muscle dihydropyridine receptors (Cav1.1) act as Ca2+ channels and voltage sensors to initiate muscle contraction by activating ryanodine receptors, the Ca2+ release channels of the sarcoplasmic reticulum. Cav1.1 activity is enhanced by a retrograde stimulatory signal delivered by the ryanodine receptor. JP45 is a membrane protein interacting with Cav1.1 and the sarcoplasmic reticulum Ca2+ storage protein calsequestrin (CASQ1).

We hypothesized that JP45 and CASQ1 form a signalling pathway which modulates Cav1.1 channel activity.

## Materials and methods

We isolated flexor digitorum brevis (FDB) muscle fibres from JP45 and CASQ1 double knock-out mice (DKO) and tested whether there were differences in Ca2+ homeostasis between the different mouse lines.

## Results

Our results show that Ca2+ transients evoked by tetanic stimulation in DKO fibres, result from massive Ca2+ influx due to enhanced Cav1.1 channel activity. This enhanced activity causes an increase of muscle strength both *in vitro* and *in vivo*.

## Conclusions

We conclude that skeletal muscle contraction is strengthened through the modulation of Cav1.1 channel activity by JP45 and CASQ1.

